# CTNS mRNA molecular analysis revealed a novel mutation in a child with infantile nephropathic cystinosis: a case report

**DOI:** 10.1186/s12882-019-1589-2

**Published:** 2019-10-31

**Authors:** Svetlana Papizh, Victoria Serzhanova, Alexandra Filatova, Mikhail Skoblov, Vyacheslav Tabakov, Lambert van den Heuvel, Elena Levtchenko, Larisa Prikhodina

**Affiliations:** 1 Department of hereditary and acquired kidney diseases, Research and Clinical Institute for Pediatrics at the Pirogov Russian National Research Medical University, 125412, Taldomskaya st., 2, Moscow, Russia; 2 Research Centre for Medical Genetics, 115522 Russia Moscow,; 3 Department of Pediatrics/Pediatric Nephrology, University Hospital Gasthuisberg, Leuven, Belgium

**Keywords:** Nephropathic cystinosis, Fanconi syndrome, *CTNS*, mRNA analysis

## Abstract

**Background:**

Cystinosis is an autosomal recessive lysosomal storage disorder characterized by accumulation of cystine in lysosomes throughout the body. Cystinosis is caused by mutations in the *CTNS* gene that encodes the lysosomal cystine carrier protein cystinosin. *CTNS* mutations result in either complete absence or reduced cystine transporting function of the protein. The diagnosis of nephropathic cystinosis is generally based on measuring leukocyte cystine level, demonstration of corneal cystine crystals by the slit lamp examination and confirmed by genetic analysis of the *CTNS* gene.

**Case presentation:**

A boy born to consanguineous Caucasian parents had the characteristic clinical features of the infantile nephropathic cystinosis including renal Fanconi syndrome (polydipsia/polyuria, metabolic acidosis, hypokalemia, hypophosphatemia, low molecular weight proteinuria, glycosuria, cystine crystals in the cornea) and elevated WBC cystine levels. Initially we performed RFLP analysis of the common in the Northern European population 57-kb deletion of proband’s DNA, then a direct Sanger sequencing which revealed no mutations in the coding part of the *CTNS* gene. To confirm the diagnosis we performed RT-PCR analysis of total RNA obtained from patient-derived fibroblasts in combination with cDNA sequencing. This revealed the skipping of exon 4 and exon 5 in the *CTNS* in our patient. Therefore, we detected a novel 9-kb homozygous deletion in the *CTNS* gene at genomic DNA level, spanning region from intron 3 to intron 5. In order to identify the inheritance pattern of the deletion we analyzed DNA of proband’s mother and father. Both parents were found to be heterozygous carriers of the *CTNS* mutation.

**Conclusions:**

Analysis of *CTNS* gene transcript allowed to identify a large homozygous deletion in the patient with infantile nephropathic cystinosis. Mutational detection at RNA level may be an efficient tool to establish the genetic defect in some cystinosis patients.

## Background

Cystinosis is an autosomal recessive lysosomal storage disorder characterized by accumulation of cystine within all organs as a result of mutations in the *CTNS* gene (MIM 606272; GenBank NM_004937.2 17p13), which encodes the lysosomal cystine transporter cystinosin [1]. The lack of functional cystinosin causes accumulation and crystallization of cystine within the lysosomes, which leads to apoptosis and tissue damage in all organs [2, 3]. Lysosomal cystine accumulation results in intracellular oxidative stress and may activate inflammasome-related gene expression in proximal tubular epithelial cells [4, 5].

The incidence of infantile nephropathic cystinosis is 1 case per 100,000–200,000 live births in the United States and Europe [6, 7]. Higher incidence rate is observed in selected populations with detected founder mutations [8]. The most frequent and most severe form of cystinosis is infantile cystinosis (MIM 219800), presenting during the first year and progressing to end-stage kidney disease (ESKD) in the first decade of life [1]. The juvenile form (MIM 219900) of cystinosis is rare and accounts for 5% of all patients with manifestation of clinical symptoms in adolescence and kidney dysfunction at variable ages, eventually leading to ESKD [9]. Non-nephropathic (ocular) cystinosis (MIM 219750) is characterized by adult-onset mild photophobia caused by cystine accumulation in the cornea of the eye without renal manifestations [10].

The *CTNS* gene consists of 12 exons with exons 3–12 being coding [11]. *CTNS* mutations result in either complete absence or reduced cystine transporting function [1, 12]. The most common mutation in the Northern European population is a large 57-kb deletion, affecting the first 10 exons of *CTNS* [12–14]. Over 140 different pathogenic *CTNS* mutations have been identified in diverse world populations, including 57 missense and nonsense mutations, 23 intronic mutations, 45 deletions, 13 small insertions, 4 indels and 3 promoter region mutations [15].

Specific treatment with orally administered cysteamine, which acts by depleting cystine in lysosomes, delays the progression of cystinosis to ESKD and postpones the occurrence of other extra-renal organ involvement, but without any effect on renal Fanconi syndrome [13, 16–18]. Cysteamine eye drops may mitigate visual symptoms by dissolving corneal cystine crystals. Supportive treatment of renal Fanconi syndrome includes providing appropriate nutrition and substituting renal losses (these are crucial to allow satisfactory growth); correcting the electrolyte and metabolic disturbances, phosphorus, vitamin D, magnesium, carnitine and calcium replacement therapy; non-hormonal anti-inflammatory agents to increased reabsorption of sodium chloride, water and decreased urine output [19].

Here we describe a patient with typical phenotype of infantile nephropathic cystinosis with elevated white blood cell (WBC) cystine levels, but initially without identified mutations in the *CTNS* gene by Restriction Fragment Length Polymorphism (RFLP) and Sanger sequencing of genomic DNA. Next we performed Real Time Polymerase Chain Reaction (RT-PCR) analysis of the CTNS mRNA transcript obtained from patient-derived fibroblasts. Based on the identified aberration of CTNS mRNA sequence we detected a novel 9-kb homozygous deletion in the *CTNS* gene and confirmed the molecular diagnosis of infantile nephropathic cystinosis in this patient.

## Case presentation

The boy was born from consanguineous Caucasian parents at 40^th^ week of pregnancy with birth weight 4100 g and height 54 cm. There was no family history of kidney disease. His growth and developmental milestones were appropriate to his age until the age of 6 months. At the age of 7 months the child presented with weight loss from 7.0 to 6.0 kg, polydipsia and polyuria (7.9 L/m^2^ per day), and developmental delay. At the age of 9 months low serum potassium (3.3 mmol/L) and phosphorus (0.59 mmol/L) levels were revealed. At first admission at the age of 12 months the boy had full-blown Fanconi syndrome including polyuria, phosphaturia with decreased ratio of tubular maximum reabsorption rate of phosphate to estimated glomerular filtration rate (TmP/GFR), glycosuria (4+), low molecular weight proteinuria with high urinary beta-2 microglobulin level (> 2.5 mg/L; normal < 0.3 mg/L), increased of fractional excretion of uric acid, potassium and sodium, aminoaciduria, metabolic acidosis, growth retardation and rickets (Table 1). His eGFR was in the normal range according to his age.

**Table 1:** Clinical data of patient

Clinical features	Results			
Age	1 y	1 y 9 mo	2 y 10 mo	3 y 11 mo
Weight, kg (‰)	6 (< 3)	7 (< 3)	11.4 (3)	13 (10)
Height, сm (‰)	68 (< 3)	74 (< 3)	84 (3)	91 (3)
Urine output, l/m^2^ (N < 2.0)	7.3	8.2	4.8	4.5
Serum				
pH (N 7.35–7.45)	7.3	7.35	7.44	7.41
HCO_3_^−^, mmol/l (N 22–26)	15.1	21.2	26	25
Sodium, mmol/l (N 135–147)	131	135	133	139
Potassium, mmol/l (N 3.7–5.1)	2.4	3.5	3.6	2.9
Chloride, mmol/l (N 97–115)	81	99	100	98
Phosphorus, mmol/l (N 1.3–2.2)	0.53	1.24	1.3	1.11
Alkaline phosphatase, IU/l (N < 644)	903	711	390	1097
Thyroid-stimulating hormone, mIU/ml (N 0.34–5.6)	5.8	5.4	5.2	4.7
WBC cystine, nmol half-cystine/mg protein (N < 1.0)		3.5	1.67	0.08
eGFR, ml/min/1.73 m^2^	77.5	54	69.7	69.2
24-h urine analysis				
Calcium/creatinine ratio, mmol/mmol (N < 1.5)	0.9	2.2	3.3	2.5
TmP/GFR, mmol/mmol (N 1.15–2.44)	0.3	0.4	0.9	0.8
Tubular urate excretion, % (N < 10%)	30	52	59	39
Tubular potassium excretion, % (N < 10%)	20	67	38	45
Tubular sodium excretion, % (N < 1%)	1.4	1.2	1.4	1.1

Kidney ultrasound revealed medullary nephrocalcinosis grade 1 (single hyperechogenic areas in the pyramids of both kidneys). The slit-lamp examination did not show any cystine crystals in the cornea. To confirm the diagnosis we performed RFLP analysis of the common in the Northern European population 57-kb deletion and direct Sanger sequencing of proband’s DNA. But no mutations in the coding part of the *CTNS* gene were detected. Therefore, nephropathic cystinosis was initially excluded, and excessive investigations were performed to search for other underlying causes of renal Fanconi syndrome such as Lowe syndrome, tyrosinemia, galactosemia, glycogenosis type 1, Wilson disease and mitochondrial diseases.

At the age of 21 months repeated slit-lamp examination revealed cystine crystals in the cornea in the boy. A cystine-binding protein assay showed a high free cystine content in leukocytes (Table 1) confirming the diagnosis of infantile nephropathic cystinosis in our patient. Oral cysteamine therapy (1.1 g/m^2^/day) and ophthalmic solution of cysteamine hydrochloride (one drop four times daily) were started.

Twelve-month therapy resulted in improvement of growth parameters, motor development (start walking independently), normalization of electrolyte disturbances and acidosis. A cystine-binding assay showed decreased WBC cystine level. Therefore, the cysteamine dose was increased to 1.2 g/m^2^/day. At the last follow-up, at the age of 4 years a cystine-binding protein assay showed normalization of WBC levels (Table 1).

Thus, the boy with phenotype of infantile cystinosis did not have mutations in the *CTNS* gene. We hypothesized that the boy might have splicing or regulatory *CTNS* mutations. To check this hypothesis we performed RT-PCR analysis of total RNA of the *CTNS* gene in the proband.

Primary fibroblasts were obtained from forearm skin biopsy as previously described by Marakhonov et al [20]. The total RNA was extracted from fibroblasts by the standard Trizol-based method. cDNA was prepared with the ImPromII Reverse Transcription System (Promega, Madison, WI) according to the manufacturer’s recommendations.

PCR analysis was performed on cDNA using primer pairs for amplifying the entire coding regions of the *CTNS* gene (Table 2). PCR fragments were analyzed by agarose gel electrophoresis in comparison with fibroblasts of a healthy donor. All abnormal fragments were sequenced by direct Sanger sequencing.

**Table 2:** Primers for PCR amplification of the *CTNS* gene

Primer	Sequence
CTNS-f1	5′-ACCTGGCGAGGCTCATGCGT-3′
CTNS-r2	5′-ACGTTGGTCGAGCTGCCGTT-3′
CTNS-f6	5′-GAAGACGTCGTTGCTGTTCAC-3’
CTNS-r6	5′-GAAGACGTCGTTGCTGTTCAC-3′
CTNS-f4	5′-GACTTCGTGGCTCTGAACCT-3’
CTNS-r4	5′-CTCCGAAGATCAGCGTCCAC-3’
CTNS-f5	5′-CAGCCTCCTGCAGATGTTCC-3’
CTNS-r1	5′-TCCTAGCCCGTCCACCAGCA-3’
CTNS-F1	5′-ATCTGGCCTCTTGCATCTCTG-3’
CTNS-R1	5′-ACATGCTCCAGCCTCAGTCTT-3’
CTNS-F2	5′-TCCCTGCTGCATGAGATCACT-3’
CTNS-R2	5′-CCATCAATGCCATCTCACACCA-3’
CTNS-F3	5′-GATCTCGGAGTGTCCAGCTAA-3’
CTNS-R3	5′-ATCTGTGGATCAGAGGGCTG-'

**Fig. 1:**
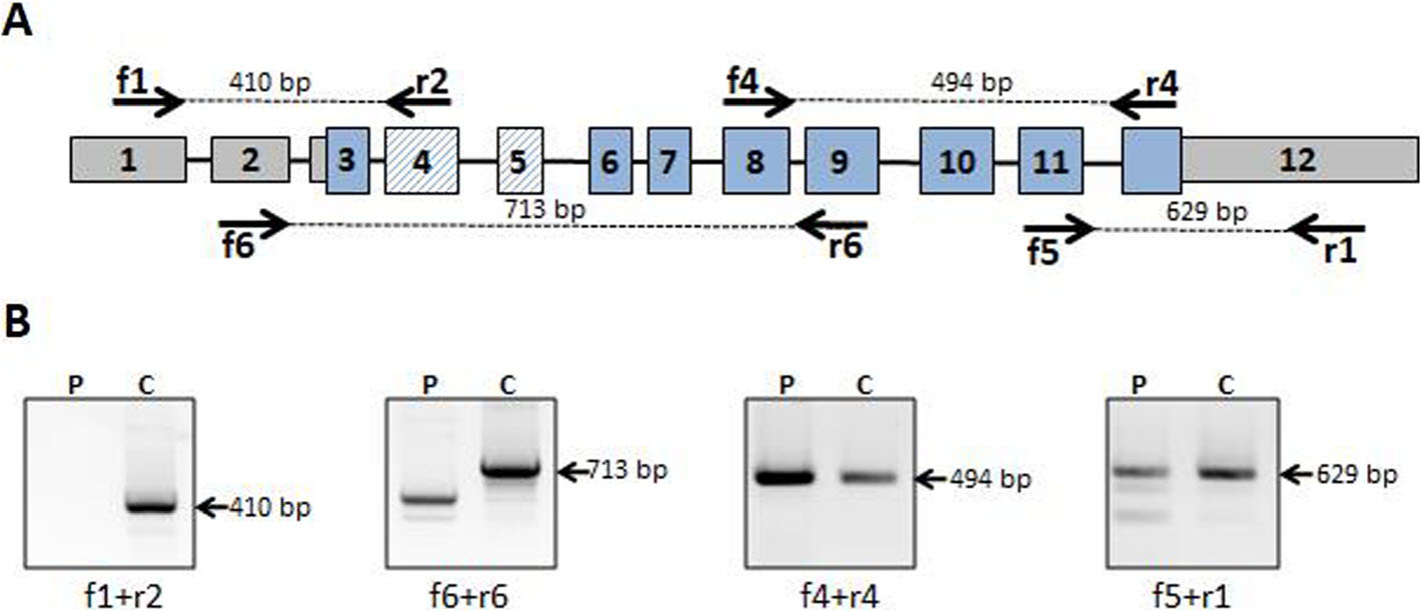
RT-PCR analysis of CTNS mRNA structure in the patient-derived fibroblasts. **a**. Scheme of the *CTNS* locus. Exons are depicted as rectangular vertical boxes. The entire coding region (CDS) is highlighted in blue. The positions of the exonic PCR primers are indicated by horizontal arrows. Primer sequences are depicted in Table 2. **b**. PCR fragments analyzed by 1% agarose gel electrophoresis. cDNA template was derived from fibroblasts: P - patient; C - control healthy donor. PCR fragment f6 + r6 is shorter than control in case of patient’s cDNA

To detect possible changes in the mRNA structure of the *CTNS* we analyzed its cDNA size and sequence in patient-derived fibroblasts by RT-PCR in combination with agarose gel electrophoresis. The entire coding region of the gene was amplified as 4 overlapping PCR fragments from cDNA template originating from the patient and healthy donor. For f6 + r6 fragment, including exon 4 and exon 5 region, we detected an abnormal size in the agarose gel compared to control (Fig. 1b). Sanger sequencing of this PCR fragment confirmed the loss of exon 4 and exon 5 in the patient’s mRNA.

**Fig. 2:**
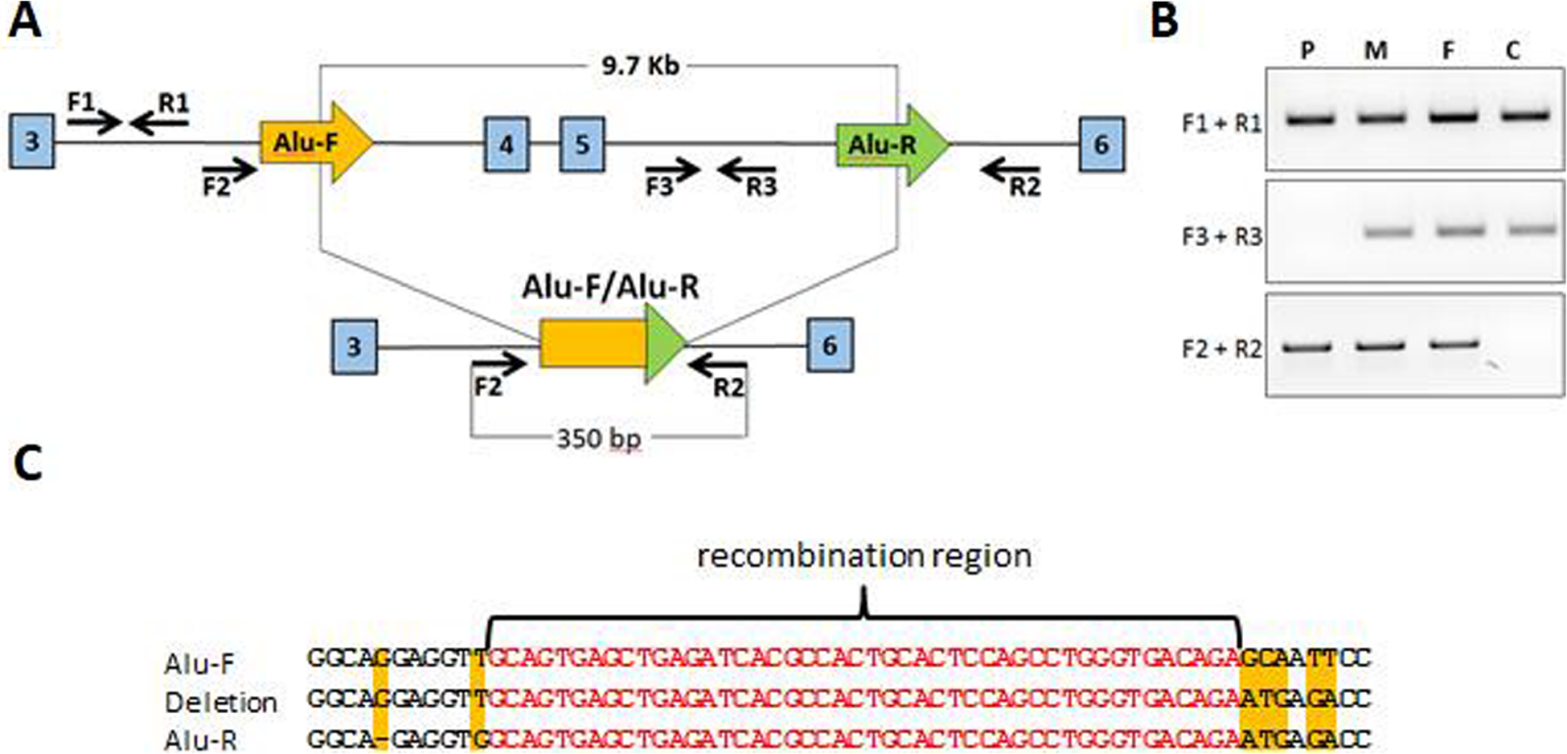
PCR analysis of the *CTNS* locus structure in the patient’s genome. **a**. Scheme of the *CTNS* locus, containing deletion. Alu sequences involved in the deletion are marked in a big arrow (Alu-F (orange) and Alu-R (green)). The positions of the intronic PCR primers are indicated by horizontal arrows. **b**. PCR fragments analyzed by 1% agarose gel electrophoresis. gDNA was used as PCR template: P - patient; M - mother; F - father; C - control healthy donor. PCR fragment F2 + R2 detected a mutated allele in 3 family members (P, M and F). PCR fragment F3 + R3 detected a WT allele in 2 carrier family members (M and F). **c**. The alignment of the recombination region. Mutated allele consists of combination of Alu-F and Alu-R sequences. The identical sequence is highlighted in red

To detect mutation region, we designed PCR primers for genomic DNA (Fig. 2a). A 9-kb homozygous deletion, spanning region from intron 3 to intron 5, was detected in patient’s DNA. In order to identify the inheritance pattern we analyzed DNA of proband’s mother and father. Both parents were found to be a heterozygous carrier (Fig. 2b).

This variant NC_000017.10:g.3545967_3555253del leads to 159 nucleotides shorter mRNA, resulting in a frameshift and truncated version of *CTNS* protein p.(Glu21GlyfsTer48). Therefore, we conclude that the new allele variant should be classified as pathogenic and was uploaded in LOVD database (ID: 0000597339).

## Discussion and conclusions

A diagnosis of infantile nephropathic cystinosis is based upon identification of characteristic symptoms, including renal Fanconi syndrome, corneal cystine crystals, a through clinical evaluation with increased WBC cystine levels, and confirmed by molecular analysis of the *CTNS* gene [21]. A prompt diagnosis of cystinosis is critical to maximize the preventive and therapeutic benefits of cystine depleting medications.

Our patient had Fanconi syndrome at the age of 7 months, but we did not find any deposition of corneal cystine crystals at the age of 1 year. Primary RFLP analysis of the common 57-kb deletion and Sanger sequencing did not reveal any pathogenic variants in the *CTNS* gene. Conventional Sanger sequence analysis can reliably detect small genetic lesions, including point mutations and small insertions/deletions, but does not detect heterozygous exonic deletions, duplications, or other rearrangements [22]. Due to this fact, we decided to perform RNA analysis as a powerful diagnostic approach to detect splice site mutations and allelic dysbalance due to regulatory mutations. Therefore, we detected a novel 9-kb homozygous deletion in the *CTNS* gene at genomic DNA level, spanning region from intron 3 to intron 5.

In general, the detection rate of *CTNS* mutations in patients with clinical diagnosis of cystinosis is ~ 95% by Sanger sequencing [15, 23]. However, Shotelersuk et al. failed to identify mutations in the *CTNS* gene in 19% of American cystinosis patients because the *CTNS* promoter was not analyzed [24]. Similar studies with analysis of the *CTNS* promoter region showed heterozygous or no mutations in 18% of Italian patients and in 6% of French patients with nephropathic cystinosis [25, 26].

According to Taranta et al. two novel mutations in the *CTNS* gene were detected by studying CTNS mRNA transcripts in patients without identified mutations in one or both alleles of the *CTNS* gene by traditional genomic sequencing [27]. Specifically, a splicing defect and DNA duplication were identified [27]. Analysis of gene transcripts is possible only in tissues with expression of this gene. Fortunately, the *CTNS* gene is expressed in various tissues and cell lines, including fibroblasts [28].

Molecular analysis of the *CTNS* gene allows not only to make an early diagnosis but also can be used for genetic counseling for the family and prenatal diagnosis of the disease. Identified mutations in the *CTNS* gene can lead to loss-of-function of the protein and manifest as the severe, infantile nephropathic phenotype as observed in the proband. On the other hand, patients with intermediate or adult forms of the disease have at least one mutation allowing the residual function of cystinosin [29]. Finding the *CTNS* mutation was important for the family of our patient because they were planning the second pregnancy. Both parents were heterozygous carriers of the same mutation (Fig. 2b) and the chance of having children with nephropathic cystinosis is 25% for each pregnancy. Prenatal diagnosis of cystinosis can be rapidly made by analysis of DNA extracted from the chorionic villi during the 1st trimester of pregnancy [23].

Genetic analysis of the *CTNS* gene is recommended for the diagnosis of nephropathic cystinosis according to the international consensus document [21]. However, in some patients with characteristic clinical features of the disease mutations in the *CTNS* gene are not detected by analysis of genomic DNA. It does not necessary exclude the diagnosis of nephropathic cystinosis in all cases and can be complemented by the examination of the CTNS mRNA transcript, which might finally establish the genetic defect in an additional number patient. Detection of mutations in the *CTNS* gene allows to early diagnosis and can be used for genetic counseling of the families.

## Data Availability

All data supporting our findings are contained within the manuscript.

## Abbreviations

DNA: Deoxyribonucleic acid
eGFR: Estimated glomerular filtration rate
ESKD: End-stage kidney disease
mRNA: Messenger ribonucleic acid
PCR: Polymerase chain reaction
RFLP: Restriction fragment length polymorphism
RT-PCR: Real time polymerase chain reaction
TmP/eGFR: Tubular maximum reabsorption rate of phosphate to estimated glomerular filtration rate
WBC: White blood cell

## References

[1] Gahl WA, Thoene JG, Schneider JA (2002). Cystinosis. N Engl J Med.

[2] Park M, Helip-Wooley A, Thoene J (2002). Lysosomal cysteine storage augments apoptosis in cultured human fibroblasts and renal tubular epithelial cells. J Am Soc Nephrol.

[3] Cherqui S, Courtoy PJ (2017). The renal Fanconi syndrome in cystinosis: pathogenic insights and therapeutic perspectives. Nat Rev Nephrol.

[4] Sumayao R, McEvoy B, Newsholme P, McMorrow T (2016). Lysosomal cystine accumulation promotes mitochondrial depolarization and induction of redox-sensitive genes in human kidney proximal tubular cells. J Physiol.

[5] Prencipe G, Caiello I, Cherqui S, Whisenant T, Petrini S, Emma F, De Benedetti F (2014). Inflammasome activation by cystine crystals: implications for the pathogenesis of cystinosis. J Am Soc Nephrol.

[6] Nesterova G, Gahl W (2008). Nephropathic cystinosis: late complications of a multisystemic disease. Pediatr Nephrol.

[7] Hult M, Darin N, von Döbeln U, Månsson JE (2014). Epidemiology of lysosomal storage diseases in Sweden. Acta Paediatr.

[8] Hutchesson AC, Bundey S, Preece MA, Hall SK, Green A (1998). A comparison of disease and gene frequencies of inborn errors of metabolism among different ethnic groups in the west midlands. UK J Med Genet.

[9] Goldman H, Scriver CR, Aaron K, Delvin E, Canlas Z (1971). Adolescent cystinosis: comparisons with infantile and adult forms. Pediatrics..

[10] Cogan DG, Kuwabara T, Kinoshita J, Sheehan L, Merola L (1957). Cystinosis in an adult. J Am Med Assoc.

[11] Town M, Jean G, Cherqui S, Attard M, Forestier L, Whitmore SA, Callen DF, Gribouval O, Broyer M, Bates GP, van t Hoff W, Antignac C (1998). A novel gene encoding an integral membrane protein is mutated in nephropathic cystinosis. Nat Genet.

[12] Kalatzis V, Antignac C (2003). New aspects of the pathogenesis of cystinosis. Pediatr Nephrol.

[13] Nesterova G, Gahl WA (2013). Cystinosis: the evolution of a treatable disease. Pediatr Nephrol.

[14] Anikster Y, Shotelersuk V, Gahl WA (1999). *CTNS* mutations in patients with cystinosis. Hum Mutat.

[15] David D, Princiero Berlingerio S, Elmonem MA, Oliveira Arcolino F, Soliman N, van den Heuvel B, Gijsbers R, Levtchenko E (2019). Molecular basis of cystinosis: geographic distribution, functional consequences of mutations in the *CTNS* gene, and potential for repair. Nephron..

[16] Gahl WA, Balog JZ, Kleta R (2007). Nephropathic cystinosis in adults: natural history and effects of oral cysteamine therapy. Ann Intern Med.

[17] Brodin-Sartorius A, Tête MJ, Niaudet P, Antignac C, Guest G, Ottolenghi C, Charbit M, Moyse D, Legendre C, Lesavre P, Cochat P, Servais A (2012). Cysteamine therapy delays the progression of nephropathic cystinosis in late adolescents and adults. Kidney Int.

[18] Cherqui S (2012). Cysteamine therapy: a treatment for cystinosis, not a cure. Kidney Int.

[19] Vaisbich MH, Satiro CAF, Roz D, Nunes DAD, Messa ACHL, Lanetzki C, Ferreira JCOA (2018). Multidisciplinary approach for patients with nephropathic cystinosis: model for care in a rare and chronic renal disease. J Bras Nefrol.

[20] Marakhonov AV, Tabakov VY, Zernov NV, Dadali EL, Sharkova IV, Skoblov MY (2018). Two novel COL6A3 mutations disrupt extracellular matrix formation and lead to myopathy from Ullrich congenital muscular dystrophy and Bethlem myopathy spectrum. Gene..

[21] Emma F, Nesterova G, Langman C, Labbé A, Cherqui S, Goodyer P, Janssen MC, Greco M, Topaloglu R, Elenberg E, Dohil R, Trauner D, Antignac C, Cochat P, Kaskel F, Servais A, Wühl E, Niaudet P, Van't Hoff W, Gahl W, Levtchenko E (2014). Nephropathic cystinosis: an international consensus document. Nephrol Dial Transplant.

[22] Feng Y, Chen D, Wang GL, Zhang VW, Wong LJ (2015). Improved molecular diagnosis by the detection of exonic deletions with target gene capture and deep sequencing. Genet Med.

[23] Levtchenko E, van den Heuvel L, Emma F, Antignac C. Clinical utility gene card for: cystinosis. Eur J Hum Genet. 2014;22(5). 10.1038/ejhg.2013.204. 10.1038/ejhg.2013.204 PMC3992566 24045844

[24] Shotelersuk V, Larson D, Anikster Y, McDowell G, Lemons R, Bernardini I, Guo J, Thoene J, Gahl WA (1998). *CTNS* mutations in an American-based population of cystinosis patients. Am J Hum Genet.

[25] Kalatzis V, Cohen-Solal L, Cordier B, Frishberg Y, Kemper M, Nuutinen EM, Legrand E, Cochat P, Antignac C (2002). Identification of 14 novel *CTNS* mutations and characterization of seven splice site mutations associated with cystinosis. Hum Mutat.

[26] Mason S, Pepe G, Dall’Amico R, Tartaglia S, Casciani S, Greco M, Bencivenga P, Murer L, Rizzoni G, Tenconi R, Clementi M (2003). Mutational spectrum of the *CTNS* gene in Italy. Eur J Hum Genet.

[27] Taranta A, Wilmer MJ, van den Heuvel LP, Bencivenga P, Bellomo F, Levtchenko EN, Emma F (2010). Analysis of *CTNS* gene transcripts in nephropathic cystinosis. Pediatr Nephrol.

[28] Jonas AJ, Greene AA, Smith ML, Schneider JA (1982). Cystine accumulation and loss in normal, heterozygous, and cystinotic fibroblasts. Proc Natl Acad Sci U S A.

[29] Kalatzis V, Nevo N, Cherqui S, Gasnier B, Antignac C (2004). Molecular pathogenesis of cystinosis: effect of *CTNS* mutations on the transport activity and subcellular localization of cystinosin. Hum Mol Genet.

